# Geographical Differences of Risk of Asthma and Allergic Rhinitis according to Urban/Rural Area: a Systematic Review and Meta-analysis of Cohort Studies

**DOI:** 10.1007/s11524-023-00735-w

**Published:** 2023-05-16

**Authors:** Mincheol Song, Seohyeon Hwang, Eunjeong Son, Hye Ju Yeo, Woo Hyun Cho, Tae Woo Kim, Kihun Kim, Dongjun Lee, Yun Hak Kim

**Affiliations:** 1 Department of Medicine, Pusan National University, Yangsan, Republic of Korea; 2 Division of Respiratory and Allergy, Department of Internal Medicine, Pusan National University Yangsan Hospital, Yangsan, Republic of Korea; 3 Department of Convergence Medicine, School of Medicine, Pusan National University, Yangsan, 50612 Republic of Korea; 4 Department of Orthopaedic Surgery, Pusan National University Yangsan Hospital, Yangsan, Republic of Korea; 5 Department of Biomedical Informatics, School of Medicine, Pusan National University, Yangsan, Republic of Korea; 6 Department of Anatomy, School of Medicine, Pusan National University, Yangsan, Republic of Korea

**Keywords:** Asthma, Allergic rhinitis, Urban, Rural, Meta-analysis, Systematic review

## Abstract

Several studies have demonstrated an association between the risk asthma/allergic rhinitis and the environment. However, to date, no systematic review or meta-analysis has investigated these factors. We conducted a systematic review and meta-analysis to assess the association between urban/rural living and the risk of asthma and allergic rhinitis. We searched the Embase and Medline databases for relevant articles and included only cohort studies to observe the effects of time-lapse geographical differences**.** Papers containing information on rural/urban residence and respiratory allergic diseases were eligible for inclusion. We calculated the relative risk (RR) and 95% confidence interval (CI) using a 2 × 2 contingency table and used random effects to pool data. Our database search yielded 8388 records, of which 14 studies involving 50,100,913 participants were finally included. The risk of asthma was higher in urban areas compared to rural areas (RR, 1.27; 95% CI, 1.12–1.44, *p* < 0.001), but not for the risk of allergic rhinitis (RR, 1.17; 95% CI, 0.87–1.59, *p* = 0.30). The risk of asthma in urban areas compared to rural areas was higher in the 0–6 years and 0–18 years age groups, with RRs of 1.21 (95% CI, 1.01–1.46, *p* = 0.04) and 1.35 (95% CI, 1.12–1.63, *p* = 0.002), respectively. However, there was no significant difference in the risk of asthma between urban and rural areas for children aged 0–2 years, with a RR of 3.10 (95% CI, 0.44–21.56, *p* = 0.25). Our study provides epidemiological evidence for an association between allergic respiratory diseases, especially asthma, and urban/rural living. Future research should focus on identifying the factors associated with asthma in children living in urban areas. The review was registered in PROSPERO (CRD42021249578).

**Supplementary Information:**

The online version contains supplementary material available at 10.1007/s11524-023-00735-w.

## Introduction

Asthma is a long-term inflammatory condition affecting the airways which causes repeated occurrences of wheezing, difficulty breathing, tightness in the chest, and/or coughing mediated by IgE antibodies and Th2 lymphocytes [1], and allergic rhinitis refers to a condition characterized by sneezing, nasal itching, difficulty in breathing due to airflow obstruction, and clear nasal discharge, resulting from an IgE-mediated response to inhaled allergens that triggers inflammation of the nasal mucosa [2]. Asthma caused 21.6 million disability-adjusted life years (DALYs), which accounted for 20% of all DALYs resulting from chronic respiratory diseases [3].

Respiratory allergic diseases, such as allergic rhinitis and asthma, are common in industrialized areas [4] and have complex pathophysiology [5, 6]. Urbanization, with its associated exposure to risk factors, has been linked to an increased risk of asthma [7, 8]. The hygiene hypothesis suggests that early exposure to infectious agents may provide protection against allergic diseases, including asthma [9]. Genetic factors play a role in asthma incidence [10], but environmental factors such as climate, urban/rural environment, diet, infant breastfeeding, smoking, pollution, obesity, and physical exercise are also important drivers of disease burden [11–17].

Recently, an increase in the incidence of respiratory allergic diseases has been reported in urbanized areas [8, 18, 19]. A systematic review and meta-analysis investigating the link between urbanization and increased asthma prevalence was published in 2019 [20]. Nonetheless, this study had a specific focus on low-income and middle-income countries, and the majority of the included studies were cross-sectional design. Therefore, our systematic review and meta-analysis aim to investigate the difference in the incidence of asthma and allergic rhinitis between urban and rural areas.

## Methods

### Patient and Public Involvement

Not applicable.

### Protocol and Registration

This systematic review was conducted in accordance with the Preferred Reporting Items for Systematic Reviews and Meta-Analyses [21, 22]. The study protocol was approved by PROSPERO.

### Eligibility Criteria

We defined the PICO statement as follows:

Participants: subjects with information on residence and asthma/allergic rhinitis

Intervention or exposure: residents of urban areas

Comparison: residents in rural areas

Outcome: difference in the incidence of asthma or allergic rhinitis

Based on this PICO statement, we conducted a search for papers that contain information on rural/urban residence and respiratory allergic diseases, specifically asthma and allergic rhinitis. Respiratory allergic diseases were defined as asthma and allergic rhinitis. Only cohort studies were eligible to observe the effects of geographical differences over time. In addition to published papers, article-in-press type papers were also considered. Only studies that clearly described respiratory allergic diseases (e.g., asthma, allergic rhinitis), and not respiratory symptoms (e.g., rhinitis, wheezing), were included. We included the most recent research in the studies using the same database.

### Information Sources and Search Strategy

On April 19, 2021, we conducted a search for papers using the Embase and Medline databases. We first considered mesh terms to establish the search strategy, and then we additionally considered text words to conduct a more extensive search. The search strategy was as follows: (rural:ab,ti OR agrarian:ab,ti OR provinc*:ab,ti OR rusti*:ab,ti OR geography*:ab,ti OR urban:ab,ti OR city:ab,ti OR municipal:ab,ti OR civil:ab,ti OR metropolitan:ab,ti) AND (respiratory tract allergy:ab,ti OR asthma:ab,ti OR bronchial hypersensitiv*:ab,ti OR bronchial hyperreacti*:ab,ti OR bronchial spasm:ab,ti OR bronchospasm:ab,ti OR bronchoconstrict*:ab,ti OR allergic rhinitis:ab,ti OR rhino conjunctivitis:ab,ti OR nasal allergy:ab,ti OR nasal hypersensitiv*:ab,ti OR nasal hyperreacti*:ab,ti OR hay fever:ab,ti) AND (risk:ab,ti OR ratio:ab,ti OR prevalence:ab,ti OR incidence:ab,ti OR outcome:ab,ti OR prognosis:ab,ti OR hazard:ab,ti OR odds:ab,ti OR morbidity:ab,ti OR cohort:ab,ti). Only papers published in English were eligible, and there were no restrictions on the publication year. We searched for gray literatures on Google and Google Scholar.

### Selection Process

The corresponding authors initially extracted records in the form of a comma-separated value (CSV) file by column. Two reviewers (MS and SH) independently screened the titles and abstracts of each study, and the full-text articles were reviewed and assessed by the same authors. Any disagreements were resolved through discussion among the authors.

### Data Collection Process and Data Items

Two reviewers (MS and SH) collected the data, which were then reviewed by the corresponding authors (KK, DL, and YHK). Any discrepancies in the data collection were resolved through consultation with the authors. Through full-text assessment, the two reviewers extracted the following data: title, abstract, author name, publication year, study period, region, number of participants, age, type of respiratory allergic disease, and classification of urban/rural areas.

### Risk of Bias Assessment

The Newcastle-Ottawa scale was used to evaluate the risk of bias in cohort studies [23]. This scale consists of eight items in three domains: selection, comparability, and outcome. Studies are defined as “good,” “fair,” and “poor” quality according to the item score. The Newcastle-Ottawa scale is one of the most widely used tools for assessing bias risk in observational studies [24]. It is a widely validated tool that allows researchers to evaluate studies across various disciplines [24]. Two reviewers (MS and SH) independently assessed the risk of bias of the included studies, and any disagreements were resolved through discussions among all authors.

### Effect Measures

The relative risk (RR) was calculated using a 2 × 2 contingency table. The RR presented in the included studies was extracted if the sample number could not be extracted. Unadjusted values were preferred, followed by adjusted values. For meta-analysis purposes, the hazard ratio (HR) was considered equal to an RR [25]. The odds ratio was converted to an RR estimate using the method described by Zhang and Kai [26].

### Data Synthesis

The heterogeneity of the included studies was evaluated using *I*^2^ statistics, with values of 25%, 50%, and 75% representing low, moderate, and high heterogeneity, respectively [27]. Since significant heterogeneity (>50%) was observed in all of our results, only the random effects model was utilized. A Higgins method calculation was used whenever an integrated value was required in the study [27]. The integrated estimate was calculated using the inverse variance weighting method [28]. Review Manager 5.4 software (Cochrane, London, UK) was used to synthesize the results, and the findings were visualized using forest plots created in the same software.

### Subgroup Analysis

For asthma, subgroup analysis was performed according to diagnostic period: 0–2 years group (until toddler), 0–6 years group (until pre-school), and 0–18 years group (until adolescent) [29], and regions such as Canada, Europe, Asia, and the USA. For allergic rhinitis, subgroup analysis was performed for 0–18 years group (until adolescent) by diagnostic period [29].

### Publication Bias

A funnel plot was drawn using the STATA 13 software (Stata Corporation, TX, USA) to visually assess publication bias. Egger’s regression test was used to quantitatively evaluate publication bias using the STATA 13 software (Stata Corporation, TX, USA).

### Certainty Assessment

Using the GRADE approach, we assessed the quality of evidence for the primary outcome based on the five required domains (study limitation, directness, consistency, precision, and reporting bias) and three additional domains (dose-response association, plausible confounding factors that would decrease the observed effect, and strength of association) [30, 31]. Two reviewers (MS and HS) independently evaluated the quality of the evidence. Any disagreements were resolved via discussions with all authors.

## Results

### Study Selection

**Fig. 1:**
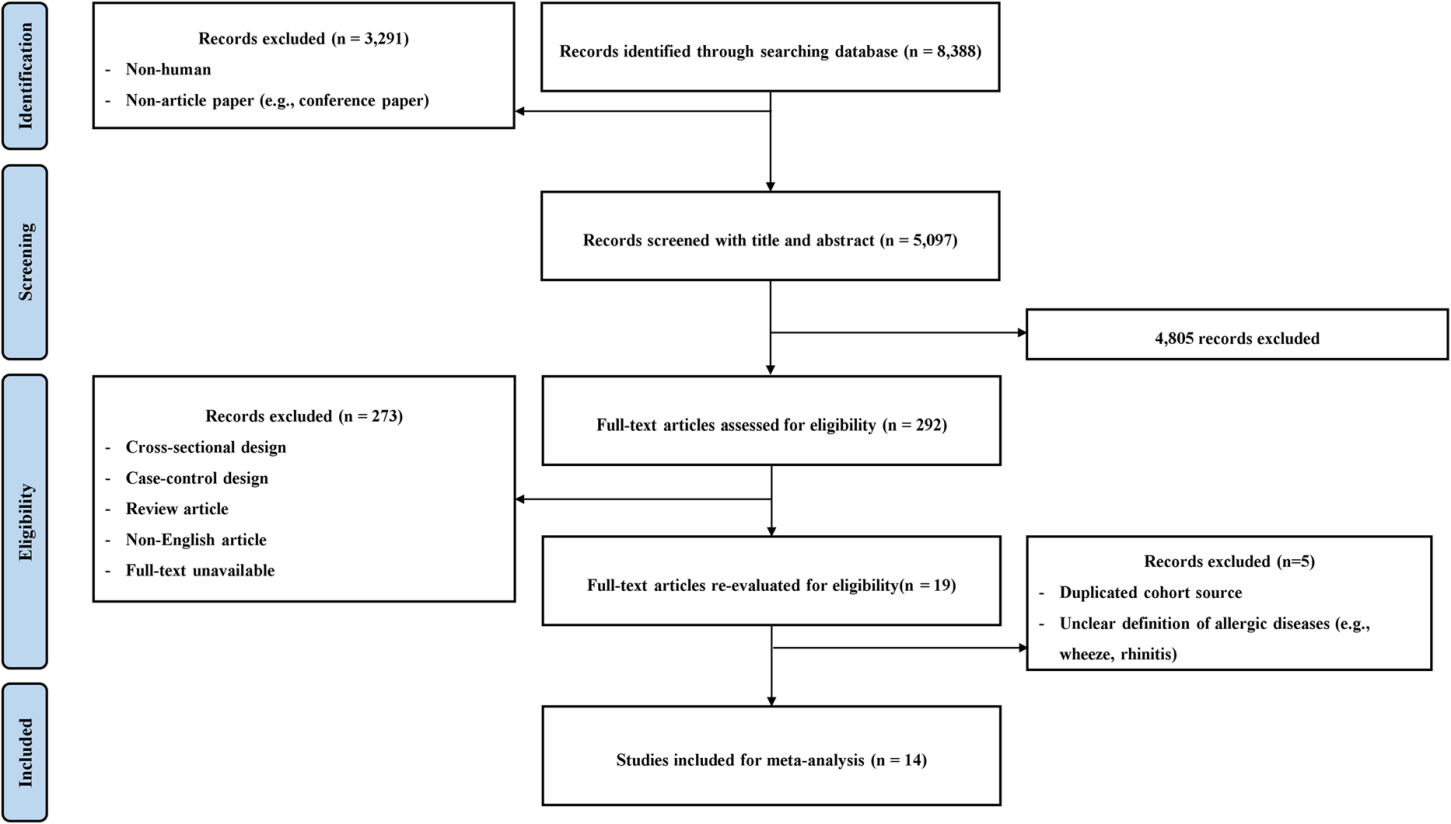
Preferred Reporting Items for Systematic Reviews and Meta-Analyses flow diagram for inclusion studies

The initial database search resulted in 8388 records. Out of these, 3291 animal studies or non-article papers were excluded, leaving 5097 records for screening. Among these, 4805 papers were excluded based on their titles and abstracts, and 292 were selected for a more detailed evaluation. Upon further evaluation, 273 papers were deemed unsuitable for analysis due to reasons such as having a case-control or cross-sectional design, being review articles, being non-English articles, or having an unavailability of full-text. After re-evaluating the 19 remaining papers, five papers were not eligible for inclusion because of duplicated cohort sources and unclear definitions of respiratory allergic diseases. Finally, 14 studies were included in the meta-analysis, as shown in Fig. 1.

### General Characteristics of the Included Studies

The characteristics of the included studies are summarized in Table 1. All included papers had cohort designs, and 50,100,913 participants from 10 countries were included in the analysis. Fourteen studies that were published between 2003 and 2020 have been analyzed. Two of these studies were conducted in the USA, four in Canada, four in Europe, and three in Asia. Thirteen of the studies were included in the asthma meta-analysis, and four were included in the allergic rhinitis meta-analysis. Most of the included studies focused on individuals younger than 20 years of age. The definition of urban/rural varied between the studies. The diagnosis of the diseases was established using questionnaires or national databases.

**Table 1:** Characteristics of the included studies

Author, year	Region	Study period	Number of samples	Age	Type of respiratory allergy	Urban/rural classification	Definition of disease	Adjusted variables	Quality assessment
Akmatov M.K., 2020 [8]	Germany	2009–2016	Urban: 32,400,372 Rural: 14,028,047	All ages	Asthma	Urban: Germany’s 16 federal states included 106 administrative districts in 2011 Rural: Germany’s 16 federal states included 296 administrative districts in 2011	ICD-10-GM, code J45	Not specified	Good
Levin M.E., 2020 [18]	South Africa	Not specified	Urban: 1185 Rural: 398	12–36 months	Asthma, AR	Urban: Cape Town Rural: Eastern Cape province	Modified ISSAC criteria	No adjustment	Poor
Norbäck D., 2018 [19]	China	2005–2012	Urban: 29,262 Rural: 1520	3–6 years	Asthma	Not specified	Yes/no question asking if the child had ever been diagnosed by a doctor	All personal factors (sex, age, birth season, birth weight, breastfeeding, parental atopy), all indoor factors (lifetime passive smoking, lifetime mold/dampness) and outdoor factors (living near traffic, lifetime outdoor T, lifetime outdoor NO_2_), and socioeconomic status (SES) factors (maternal occupation during pregnancy, home size, number of persons living in the current home, current residential area, and GDP per capita)	Poor
Lavin T., 2017 [32]	India, Vietnam	2002–2016	Urban: 833 Rural: 2969	7.5–8.5 years	Asthma	Several factors are considered by two reports to assess whether a community/environment type reflects a rural or urban environment, including agriculture and industry, and environment and services	Face-to face interviews if the child had long-term respiratory problems such as asthma (may include clinical diagnosis or hospital admission)	No adjustment	Poor
Dostál M., 2014 [33]	Czech Republic	1994–1999	Urban: 466 Rural: 455	0–10 years	AR	Urban: the Teplice district—can produce highly concentrated pollutants from both local and remote sources Rural: the less polluted rural district of Prachatice	ICD-10 codes	Sex, low birth weight (< 2500 g) and/or gestation < 37 weeks, maternal education, maternal allergy, an older sibling with allergy, and breastfeeding	Good
Lawson J.A., 2014 [34]	Canada	1994–2007	Urban: 1,649,462 Rural: 389,428	12–18 years	Asthma	Areas with a population of fewer than 1000 people or with a population density of <400 people/km^2^ were considered rural based on Canadian Census Rural Areas which accounts for population size and density	Has [NAME] ever had asthma that has been diagnosed by a health professional?	Sex, age, income, body mass index, passive smoking exposure, personal smoking, physical exercise	Good
Stoner A.M., 2013 [11]	USA	2002–2006	Urban: 5800 Rural: 1100	5.5 years	Asthma	Not specified	Since your child turned (x) years of age, has a doctor, nurse, or other medical professional ever told you that your child has asthma?	No adjustment	Good
Valet R.S., 2011 [35]	USA	1995–2000	Urban: 52,168 Rural: 38,317	0–5.5 years	Asthma, AR	Urban: central county within 1 of Tennessee’s 4 standard metropolitan statistical areas Rural: county outside a standard metropolitan statistical areas	ICD-9 codes	Birth weight, sex, race, chronic medical conditions, history of bronchiolitis, maternal smoking, maternal education, and maternal history of asthma	Good
Midodzi W.K., 2010 [7]	Canada	1996–2003	Urban: 5823 Rural: 2602	<2 years	Asthma	Urban: central metropolitan area Rural: non-central metropolitan area	Physician-diagnosed	Sex, birth weight, breastfeeding, history of early wheezing, history of childhood allergy, history of nose/throat infection, early daycare attendance, single parent household, maternal medication use in pregnancy, maternal smoking during pregnancy, number of older siblings present at birth, household socio-economic index, and geographic regions	Poor
Midodzi W.K., 2007 [36]	Canada	1994–1997	Urban: 10,945 Rural: 2570	0–11 years	Asthma	Not specified	Physician-diagnosed	Age, sex, child allergies, no. of pediatrician visits, no. of physician visits, mothers’ age at child’s birth, older sibling, parental history of asthma, immigrant mother, either parent smoked, home needing repairs, geographic region, body mass index, socioeconomic status, and crowding index	Poor
Priftis K.N., 2007 [37]	Greece	1995–2004	Urban: 446 Rural: 312	8–10 years	Asthma	Urban: municipality of Maroussi characterized by the air pollution with heavy traffic Rural: Aliartos and another three adjoining agricultural villages 5 km around the station monitoring air quality in the area	The questionnaire included the ISAAC core questions on symptoms of asthma, physician-diagnosed asthma	No adjustment	Poor
Bråbäck L., 2004 [38]	Sweden	1952–1981	Urban:1,119,437 Rural: 197,548	17–20 years	Asthma, AR	Urban is defined as a home located in a settlement with at least 200 inhabitants.	ICD-8, 9, and 10 codes	County of residence, urban/rural living, family size, overcrowding, being the first-born boy, and maternal age at the birth of the child	Good
Dik N., 2004 [39]	Canada	1980–1990	Urban: 76,472 Rural: 81,118	0–6 years	Asthma	Urban: metropolitan Winnipeg—approximately 650,000 or 54% of the total provincial population Rural: outside Winnipeg—the largest town having 30,000 people	ICD-493	Sex, primary care	Good
Shima M., 2003 [12]	Japan	1990–1997	Urban: 1020 Rural: 838	6–9 years	Asthma	Urban: six schools in four communities (Chiba, Funabashi, Kashiwa, and Ichikawa) Rural: four schools in four communities (Ichihara, Tateyama, Mobara, and Kisarazu)	Two or more episodes of wheezing accompanied by dyspnea that had ever been given the diagnosis of asthma by a physician and the occurrence of asthmatic attacks or the need for any medication for asthma during the past 2 years	Sex, school grade, history of allergic diseases, respiratory diseases before 2 years of age, breastfeeding in infancy, parental history of allergic diseases, maternal smoking habits, house of steel or reinforced concrete, use of unvented heater in winter	Good

*AR* allergic rhinitis, *ISAAC* International Study of Asthma and Allergies in Children

### The Risk of Developing Asthma and Allergic Rhinitis in Urban Residents Compared to Rural Residents

**Fig. 2:**
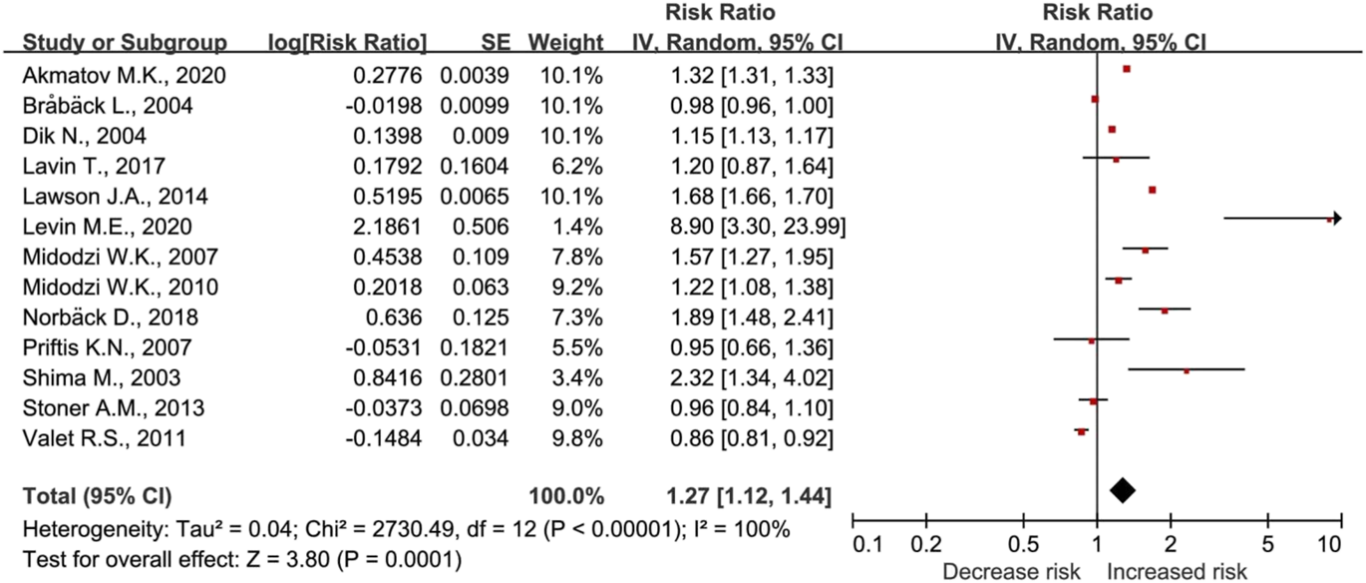
Risk of asthma in urban residents compared to rural residents

**Fig. 3:**
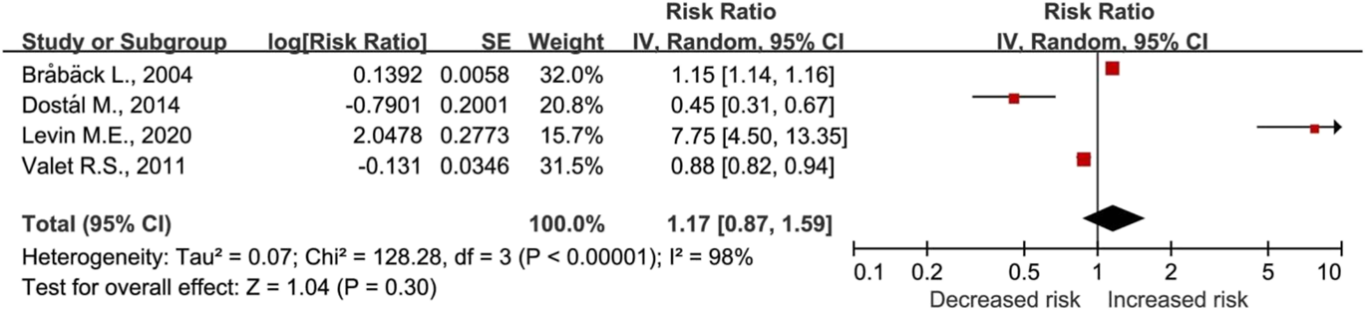
Risk of allergic rhinitis in urban residents compared to rural residents

The main and subgroup analysis results of asthma and allergic rhinitis risk are presented in Table 2. The risk of asthma was higher in urban areas compared to rural areas (RR, 1.27), but not for the risk of allergic rhinitis (RR, 1.17) (Fig. 2, Fig. 3). The risk of asthma in urban areas compared to rural areas was higher in the 0–6 years and 0–18 years age groups. However, there was no significant difference in the risk of asthma between urban and rural areas for children aged 0–2 years. The study compared the RRs of asthma in urban areas to those in rural areas across the USA, Canada, Europe, and Asia. The results showed that RRs were 0.90, 1.39, 1.09, and 1.68, for the respective regions. Furthermore, the analysis of the 0–18 years age group showed a RR of 1.42 for allergic rhinitis in urban areas compared to rural areas.

**Table 2:** Main and subgroup analysis of the risk of asthma and allergic rhinitis in urban compared to rural areas

Outcome	Number of studies (***n***)	Heterogeneity (%)	Relative risk (95% confidence interval, *p* value)
Asthma	13	100	1.27 (1.12–1.44, *p* < 0.001)
0–2 years	2	93	3.10 (0.44–21.56, *p* = 0.25)
0–6 years	6	95	1.21 (1.01–1.46, *p* = 0.04)
0–18 years	11	99	1.35 (1.12–1.63, *p* = 0.002)
USA	2	51	0.90 (0.81–0.99, *p* = 0.04)
Canada	4	100	1.39 (1.06–1.81, *p* = 0.02)
Europe	4	100	1.09 (0.85–1.41, *p* = 0.50)
Asia	3	100	1.68 (1.16–2.42, *p* = 0.006)
Allergic rhinitis	4	98	1.17 (0.87–1.59, *p* = 0.30)
0–18 years	3	97	1.42 (0.46–4.39, *p* = 0.55)

### Risk of Bias within Studies

Of the 14 studies, 8 were evaluated as “good” and 6 as “poor.” The detailed risk of bias in the included studies is shown in Supplementary Table 1. For studies identified as poor quality, either exposure or disease identification was not clear, or the adequacy of cohort follow-up was not specified.

### Publication Bias

**Fig. 4:**
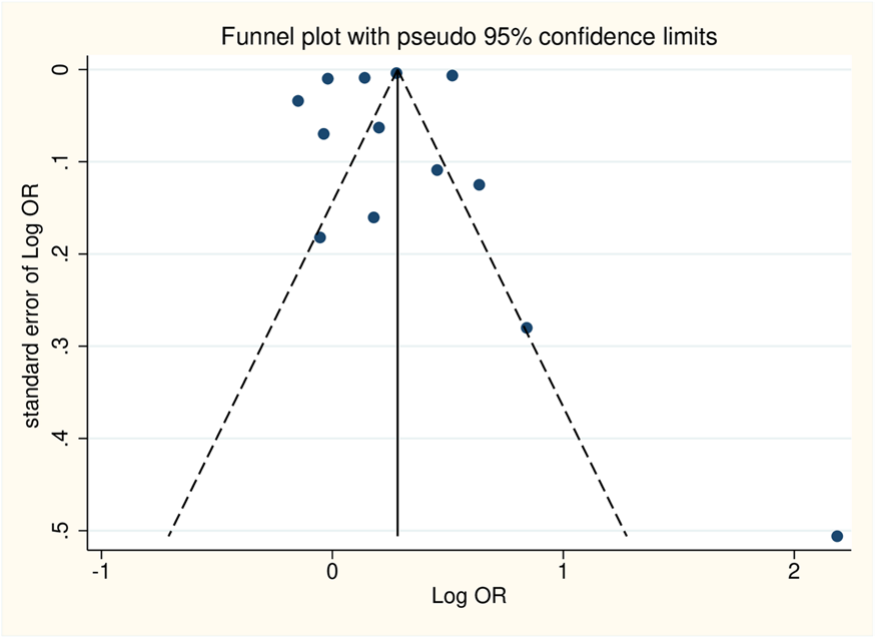
A funnel plot for risk of asthma

A funnel plot was constructed for the risk of asthma (Fig. 4). No significant publication bias was observed according to the Egger’s regression test (*p* = 0.635).

### Certainty Assessment

The certainty assessment for the eight domains was rated for risk of asthma and allergic rhinitis. The quality of evidence was evaluated as “low” for asthma and “very low” for allergic rhinitis according to the GRADE approach (Table 3). This evaluation was prompted by the inclusion of observational studies, significant heterogeneity in the results, and low directness of the evidence level, although the precision of the results attributable to the inclusion of numerous subjects. Moreover, no item was identified to enhance the rating in additional domains.

**Table 3:** GRADE approach for the primary outcome

Outcome	Quality assessment								
	Required domains					Additional domains			Grade
	Study limitations	Consistency	Directness of evidence	Precision	Reporting bias	Dose-response association	Plausible confounding that would decrease the observed effect	Strength of association (magnitude of effect)	
Asthma	High^a^	Inconsistent^b^	Indirect	Precise^c^	Undetected^d^	Undetected	Present^e^	Weak^f^	Low
Allergic rhinitis	High^a^	Inconsistent^b^	Indirect	Precise^c^	Unestimable^g^	Undetected	Present^e^	Weak^f^	Very low

^a^All included studies are observational in design

^b^Considerable heterogeneity (*I*^2^ > 50%)

^c^Very large sample size (over 4000)

^d^According to Egger’s regression test (*p* = 0.578)

^e^All included studies had an observational design, and there was a difference in adjusted variables depending on each study

^f^OR < 2.0

^g^Due to insufficient included studies to evaluate publication bias

## Discussion

Our meta-analysis systemically confirmed that urban residents had a higher risk of developing asthma than rural residents. Previous reports have indicated a global increase in asthma since the 1970s [40], with a total of 340 million individuals worldwide currently diagnosed with asthma, and an additional 100 million individuals expected to be affected by 2025 [41]. The prevalence of asthma tends to be higher in developed countries [42], with prevalence estimates of 3–12% among children and 2–5% among adults in Germany [43]. There has also been a considerable increase in asthma in China, and a correlation has been observed between GDP per capita at the city level and the prevalence of asthma [44]. In the USA, urban children are well understood to represent a group with high asthma prevalence [45], but little information about the burden of asthma among rural children is available, even though 21% of the population of the USA lives in rural areas [46]. Previous studies have shown that the prevalence of asthma is highly associated with poverty, independent of race [47, 48]. In particular, rural residents in the USA tend to have lower incomes, lower educational attainment, higher rates of uninsurance, and lower physician supply. A high asthma prevalence (28%) was found in a rural, poor, predominantly African American cohort of children in Arkansas [49].

There are several potential risk factors for a high prevalence of respiratory diseases in urban environments [16]. The increased risk of asthma in urban area may be partly explained by indoor and outdoor air pollution, low accessibility to greenery, and low biodiversity [50]. Several studies have demonstrated that outdoor air pollution, including NO_2_, SO_2_, and particulate matter, is a risk factor for asthma and allergic diseases [11–14], with most studies focusing on traffic-related air pollution as a risk factor for asthma [12, 15]. In addition, as the urban-rural difference in asthma prevalence is reported, a “hygiene hypothesis” has been proposed, suggesting that early exposure to the infectious or microbial environment may play a protective role against respiratory allergic diseases [37, 38].

We speculate that another possible factors for the lower prevalence of asthma in rural environments could be a lack of awareness of the condition, which may be due to lower education levels and limited access to medical care and diagnosis compared to urban areas [51]. Urban areas may have a higher influx of migrants, leading to demographic differences between urban and rural populations [52]. In addition, genetic variations between urban and rural populations within each country may differ and could also contribute to the urban/rural differences in the incidence of asthma [53].

Subgroup analysis results by region (USA, Canada, Europe, Asia) showed different trends. These differences can be explained by different levels of urbanization, different dimensions of the urban environment, and differences in living conditions among populations [20]. It is also possible that this is due to differences in the characteristics of the study subjects included or the urban-rural definition.

One of the strengths of our study was that we included subgroup studies based on asthma and allergic rhinitis. The immune system’s shift towards a pro-allergenic Th2 response due to reduced microbial exposure in early life is responsible for the Th1/Th2 cell imbalance, resulting in the clinical manifestation of asthma [54]. A recent study highlighted the major contributions of Th2 cytokines, such as IL4, IL13, and IL5, to asthma, while interferon-γ, a Th1 cytokine, has recently been shown to maintain the chronic inflammatory response in asthma. During the early phase, allergen-specific T-cells are activated and play a central role in the development of allergic asthma [55]. Studies have shown that up to 80% of childhood and 60% of adult asthma cases are linked to an unwarranted Th2 cell response to respiratory allergens [56]. Therefore, targeting Th2 modulators with neutralizing antibodies may be a promising option for asthma patients.

The limitation of our study is that we included studies with high heterogeneity, including differences in criteria for classifying urban and rural areas in each included study. Because we only included articles published in English, the results may be biased. The statistical power of allergic rhinitis results is relatively low due to the small number of studies related to allergic rhinitis. Despite these limitations, it is important to note that our findings showed a significantly higher incidence of asthma among urban residents.

## Conclusion

The findings of this study offer epidemiological evidence of an association between asthma and urban/rural residency. Our analysis revealed that urban inhabitants have a greater likelihood of developing asthma than rural residents, although no statistically significant difference was found for allergic rhinitis. Additional investigation is necessary to examine the factors linked to asthma in children who live in urban settings.

## Supplementary information

ESM 1

## Data Availability

Data sharing is not applicable to this article, as no datasets were generated or analyzed in this study.
