# Supplementary material for: Geographical Differences of Risk of Asthma and Allergic Rhinitis according to Urban/Rural Area: a Systematic Review and Meta-analysis of Cohort Studies
Source: J Urban Health. 2023 May 16;100(3):478–92. doi: 10.1007/s11524-023-00735-w (PMC10323063; doi:10.1007/s11524-023-00735-w)
Supplement: Supplementary file 1 — ESM 1 [file 11524_2023_735_MOESM1_ESM.docx]

**Supplementary text**

**Newcastle-Ottawa Scale – Cohort studies^1^**

Note: A study can be awarded a maximum of one star for each numbered item within the selection and outcome categories. A maximum of two stars may be assigned for comparability.

**Selection:** (Maximum 4 stars)

1) Representativeness of the exposed cohort

a) truly representative of the average population in the target country or region*

b) somewhat representative of the average population in the target country*

c) selected group of users

d) no description of the derivation of the cohort

2) Selection of the non-exposed cohort

a) drawn from the same community as the exposed cohort*

b) drawn from a different source

c) no description of the derivation of the non-exposed cohort

3) Ascertainment of exposure

a) secure record*

b) structured interview*

c) written self-report

d) no description

4) Demonstration that the outcome of interest was not present at the start of the study

a) yes*

b) no

**Comparability:** (Maximum 2 stars)

1) Comparability of cohorts based on the study design or analysis

a) study controls for age

b) study controls for other demographic factors*

**Outcome:** (Maximum 3 stars)

1) Assessment of outcome

a) independent blind assessment*

b) record linkage*

c) self-report

d) no description

2) Was follow-up long enough for outcomes to occur?

a) ≥1 year *

b) <1 year

3) Adequacy of follow-up of cohorts

a) complete follow-up - all subjects accounted for*

b) Subjects lost to follow-up unlikely to introduce bias–small number lost–70% follow-up, or description provided of those lost)*

c) follow-up rate < 70% and no description of those lost

d) no statement

**Supplementary Table 1: Risk of bias assessments of the included studies using the Newcastle–Ottawa Scale**

| **Study** | **Selection** | | | | **Comparability based on the design and analysis** | **Outcome** | | | **Quality Assessment*** |
| --- | --- | --- | --- | --- | --- | --- | --- | --- | --- |
|  | **Representativeness of the sample** | **Selection of the non-intervention cohort** | **Ascertainment of exposure** | **Demonstration that the outcome of interest was not present at the start of the study** |  | **Assessment of outcome** | **Was follow-up long enough for outcomes to occur?** | **Adequacy of follow-up of cohorts** |  |
| Akmatov M.K., 2020^2^ | 1 | 1 | 1 |  | 2 | 1 | 1 | 1 | Good |
| Levin M.E., 2020^3^ |  | 1 | 1 |  |  |  | 1 |  | Poor |
| Norbäck D., 2018^4^ | 1 | 1 |  | 1 | 2 |  | 1 |  | Poor |
| Lavin T., 2017^5^ | 1 | 1 |  |  | 2 |  | 1 |  | Poor |
| Dostál M., 2014^6^ |  | 1 | 1 | 1 | 2 | 1 | 1 |  | Good |
| Lawson J.A., 2014^7^ | 1 | 1 | 1 |  | 2 |  | 1 | 1 | Good |
| Stoner A.M., 2013^8^ | 1 | 1 |  | 1 | 2 | 1 | 1 | 1 | Good |
| Valet R.S., 2011^9^ | 1 | 1 | 1 | 1 | 2 | 1 | 1 |  | Good |
| Midodzi W.K., 2010^10^ | 1 | 1 |  | 1 | 2 |  | 1 |  | Poor |
| Midodzi W.K., 2007^11^ | 1 | 1 |  | 1 | 2 |  | 1 |  | Poor |
| Priftis K.N., 2007^12^ |  | 1 | 1 |  |  |  | 1 |  | Poor |
| Bråbäck L., 2004^13^ | 1 | 1 | 1 |  | 2 | 1 | 1 |  | Good |
| Dik N., 2004^14^ | 1 | 1 | 1 |  | 2 | 1 | 1 | 1 | Good |
| Shima M., 2003^15^ |  | 1 | 1 | 1 | 2 | 1 | 1 | 1 | Good |

***** Good quality:3 or 4 stars in the selection domain AND 1 or 2 stars in the compatibility domain AND 2 or 3 stars in the outcome/exposure domain; fair quality:2 stars in the selection domain AND 1 or 2 stars in the comparability domain AND 2 or 3 stars in the outcome/exposure domain; poor quality:0 or 1 star in the selection domain OR 0 stars in the comparability domain OR 0 or 1 stars in the outcome/exposure domain

**References**

**1.** Peterson J, Welch V, Losos M, Tugwell P. The Newcastle-Ottawa scale (NOS) for assessing the quality of nonrandomised studies in meta-analyses. *Ottawa: Ottawa Hospital Research Institute.* 2011.

**2.** Akmatov MK, Holstiege J, Steffen A, Bätzing J. Trends and regional distribution of outpatient claims for asthma, 2009–2016, Germany. *Bulletin of the World Health Organization.* 2020;98(1):40.

**3.** Levin ME, Botha M, Basera W*, et al.* Environmental factors associated with allergy in urban and rural children from the South African Food Allergy (SAFFA) cohort. *Journal of Allergy and Clinical Immunology.* 2020;145(1):415-426.

**4.** Norbäck D, Lu C, Wang J*, et al.* Asthma and rhinitis among Chinese children—indoor and outdoor air pollution and indicators of socioeconomic status (SES). *Environment international.* 2018;115:1-8.

**5.** Lavin T, Franklin P, Preen DB. Association between caesarean delivery and childhood asthma in India and Vietnam. *Paediatric and perinatal epidemiology.* 2017;31(1):47-54.

**6.** Dostál M, Prucha M, Rychlíková E, Pastorková A, Srám RJ. Differences between the spectra of respiratory illnesses in children living in urban and rural environments. *Central European Journal of Public Health.* 2014;22(1):3.

**7.** Lawson JA, Janssen I, Bruner MW, Hossain A, Pickett W. Asthma incidence and risk factors in a national longitudinal sample of adolescent Canadians: a prospective cohort study. *BMC pulmonary medicine.* 2014;14(1):1-9.

**8.** Stoner AM, Anderson SE, Buckley TJ. Ambient air toxics and asthma prevalence among a representative sample of US kindergarten-age children. *PLoS One.* 2013;8(9):e75176.

**9.** Valet RS, Gebretsadik T, Carroll KN*, et al.* High asthma prevalence and increased morbidity among rural children in a Medicaid cohort. *Annals of Allergy, Asthma & Immunology.* 2011;106(6):467-473.

**10.** Midodzi WK, Rowe BH, Majaesic CM, Saunders LD, Senthilselvan A. Early life factors associated with incidence of physician-diagnosed asthma in preschool children: results from the Canadian Early Childhood Development cohort study. *Journal of Asthma.* 2010;47(1):7-13.

**11.** Midodzi WK, Rowe BH, Majaesic CM, Senthilselvan A. Reduced risk of physician‐diagnosed asthma among children dwelling in a farming environment. *Respirology.* 2007;12(5):692-699.

**12.** Priftis KN, Anthracopoulos MB, Nikolaou‐Papanagiotou A*, et al.* Increased sensitization in urban vs. rural environment–rural protection or an urban living effect? *Pediatric allergy and immunology.* 2007;18(3):209-216.

**13.** Bråbäck L, Hjern A, Rasmussen F. Trends in asthma, allergic rhinitis and eczema among Swedish conscripts from farming and non‐farming environments. A nationwide study over three decades. *Clinical & Experimental Allergy.* 2004;34(1):38-43.

**14.** Dik N, Tate RB, Manfreda J, Anthonisen NR. Risk of physician-diagnosed asthma in the first 6 years of life. *Chest.* 2004;126(4):1147-1153.

**15.** Shima M, Nitta Y, Adachi M. Traffic-related air pollution and respiratory symptoms in children living along trunk roads in Chiba Prefecture, Japan. *Journal of epidemiology.* 2003;13(2):108-119.
